# Comparison between modified facet joint fusion and posterolateral fusion for the treatment of lumbar degenerative diseases: a retrospective study

**DOI:** 10.1186/s12893-022-01468-4

**Published:** 2022-01-28

**Authors:** Zhimin Li, Zheng Li, Xin Chen, Xiao Han, Kuan Li, Shugang Li

**Affiliations:** 1 Department of Orthopedics, Peking Union Medical College Hospital, Chinese Academy of Medical Sciences and Peking Union Medical College, No.1 Shuaifuyuan Hutong, Dong Cheng District, Beijing, 100730 China; 2 Department of Neurosurgery, Peking Union Medical College Hospital, Chinese Academy of Medical Sciences and Peking Union Medical College, No.1 Shuaifuyuan Hutong, Dong Cheng District, Beijing, 100730 China

**Keywords:** Modified facet joint fusion, Posterolateral fusion, Lumbar degenerative disease, Oswestry disability index, Surgical treatment

## Abstract

**Objective:**

To investigate the safety and effectiveness of modified facet joint fusion in the treatment of lumbar degenerative diseases and compare them with those of posterolateral fusion.

**Methods:**

A total of 77 adult patients with lumbar degenerative disease diagnosed from January 2017 to February 2019 were considered for the present retrospective, nonrandomized, and controlled study. The patients were divided into two groups according to the fusion technique used during the surgery: the posterolateral fusion (PLF) group (n = 42) and the modified facet joint fusion (MFF) group (n = 35). The fusion rate, Oswestry Disability Index (ODI) score, visual analog scale (VAS) score for back pain and leg pain, Japanese Orthopedic Association (JOA) score, European Quality of Life–5 Dimensions (EQ-5D) score, length of hospital stay, length of operation, intraoperative blood loss, cost of hospitalization, complications and reoperations were compared between the 2 groups.

**Results:**

All patients underwent a successful surgery, and all were followed up. No significant differences were found in age, sex, BMI, length of hospital stay, length of operation or cost of hospitalization. There were no significant differences in the preoperative or postoperative ODI or in the VAS, JOA, and EQ-5D scores between the MFF and PLF groups. However, the fusion rate of MFF group was higher than that of the PLF group (P < 0.05). What’s more, the MFF group had less intraoperative blood loss than the PLF group (P < 0.05). Complications related to iatrogenic nerve injury, vascular injury, epidural hematoma, intravertebral infection, and internal fixation did not occur in either group. None of the patients required reoperation.

**Conclusions:**

Modified facet joint fusion is safe and efficient in the treatment of lumbar degenerative disease. The fusion rate of MFF was higher than PLF. The intraoperative blood loss of MFF was less than that of PLF. In addition, the therapeutic effect of MFF was not worse than that of PLF. Therefore, the MFF technique can be promoted in clinical treatment.

## Introduction

With the aggravation of aging, the incidence of lumbar degenerative disease increases gradually. Lumbar fusion is an important surgical technique for the treatment of this disease [1].

Lumbar fusion is widely used in the treatment of a variety of degenerative lumbar diseases, and successful fusion is closely related to the outcome of the operation [2, 3]. Postoperative pseudarthrosis formation can lead to obvious clinical symptoms such as low back pain and often requires reoperation, but spinal surgeons have classically struggled with this condition [4, 5]. Therefore, to reduce the incidence of pseudarthrosis, various fusion techniques have been applied in lumbar surgery. Among them, posterolateral lumbar fusion (PLF), posterior lumbar interbody fusion (PLIF) and transforaminal lumbar interbody fusion (TLIF) are the most commonly used [6–9].

PLF is the most widely used fusion method in treating lumbar degenerative disease [10, 11]. Due to the need to expose the bilateral transverse processes, PLF is characterized by heavier trauma, a larger amount of bone grafting, and a lower fusion rate than other fusion techniques [12]. However, modified facet joint fusion (MFF) requires only a small fusion distance, which means that a higher fusion rate and lighter trauma can be theoretically achieved [11]. TLIF and PLIF have relatively high technical requirements, heavier trauma and higher risk of nerve injury than the other surgery techniques [13–15].

To the best of our knowledge, there are few studies on facet joint fusion. Considering the low fusion rate of PLF technology and the disadvantages of T/PLIF fusion technology, we believe that facet joint fusion technology may be an ideal choice. In this study, MFF was introduced and evaluated for its safety and effectiveness.

## Methods

### General data

A retrospective analysis of 77 adult patients with lumbar degenerative diseases treated by surgery was conducted between January 2017 and February 2019 at Peking Union Medical College Hospital. All patients were from China and of Asian ethnicity. All patients underwent preoperative lumbar X-ray (anterior and lateral X-ray, anterior flexion and posterior extension X-ray), lumbar spine CT scan, and MRI of the lumbar spine to make a definite diagnosis. Of these patients, 42 underwent posterolateral lumbar fusion and were assigned to the PLF group, and 35 underwent modified facet joint fusion and were assigned to the MFF group. In the PLF group, there were 19 males and 23 females, with an age range of 23–78 years (average age, 45.9 ± 14.1 years). In the MFF group, there were 18 males and 17 females, with an age range of 26–75 years (average age, 43.1 ± 18.4 years). The general patient data for both groups are shown in Table 1. This retrospective study was approved by the Ethics Committee of Peking Union Medical College Hospital. All methods were carried out in accordance with institutional guidelines and regulations. Due to the retrospective nature of the study, the need for informed consent was waived by the Ethics Committee of Peking Union Medical College Hospital.

**Table 1:** Baseline characters of the patients

Characteristic	PLF group (N = 42)	MFF group (N = 35)	P value	Statistical power
Age, (year)	45.86 ± 14.08	43.09 ± 18.42	0.513	0.176
Female sex, no. (%)	23 (55)	17 (49)	0.588	0.731
BMI	24.93 ± 3.38	25.37 ± 4.12	0.425	0.125
ODI score	28.43 ± 13.82	24.00 ± 14.55	0.536	0.373
VAS score for lumbar pain	5.00 ± 3.44	4.55 ± 3.24	0.767	0.141
VAS score for leg pain	5.71 ± 3.65	5.18 ± 3.03	0.572	0.164
JOA score	11.86 ± 3.74	11.91 ± 2.77	0.851	0.057
EQ-5D score	0.51 ± 0.27	0.56 ± 0.27	0.647	0.194

### Inclusion and exclusion criteria

The inclusion criteria were the ability to consent, age between 18 and 80 years old, primary lumbar fusion performed in Peking Union Medical College Hospital, fusion range of 1–2 segments, diagnosis of lumbar spinal stenosis or lumbar disc herniation and ASA level of I to II. The exclusion criteria included revision surgery, lumbar spondylolysis, lumbar spondylolisthesis above grade I, scoliosis, lumbar fracture, infection, tumor or other diseases.

### Surgical procedure

L4–5 single segment surgery will be used as an example to describe modified facet joint fusion. A midline incision was made at the lower back. The bilateral paravertebral muscles were stripped along the subperiosteum to expose the spinous process, bilateral lamina, facet joint and roots of the transverse processes of the surgical segment. Pedicle screw placement and rod manipulation were completed at the responsible segment, and C-arm fluoroscopy was performed to confirm the position and length of each screw. The lower 2/3 of the L4 lamina and the upper 1/4 of the L5 lamina were removed, and the ligamentum flavum of the L4–5 segment was resected. The medial 2/3 of the bilateral inferior articular process of L4 and the medial 1/3 of the bilateral superior articular process of L5 were removed, and the lateral 2/3 were reserved for facet joint bone grafting. After decompression or discectomy, a bone graft bed was made with the following procedure. A high-speed grinding drill was used (150 W, 30,000/min, AESCULAP INTERNATIONAL GMBH) to carefully grind the articular surface of the bilateral L4–5 facet joints to create the bone graft bed. The allogeneic cancellous bone granules and the autologous cancellous bone were carefully implanted into this bed.

For the PLF group, the inferior articular process of L4 and the medial side of the superior articular process of L5 were removed. After pedicle screw placement, a bilateral interarticular process bone graft bed of L4–L5 was made, and then bilateral interarticular process bone grafting of L4–L5 was performed with autologous cancellous bone and allogeneic cancellous bone granules.

### Evaluation

**Fig. 1:**
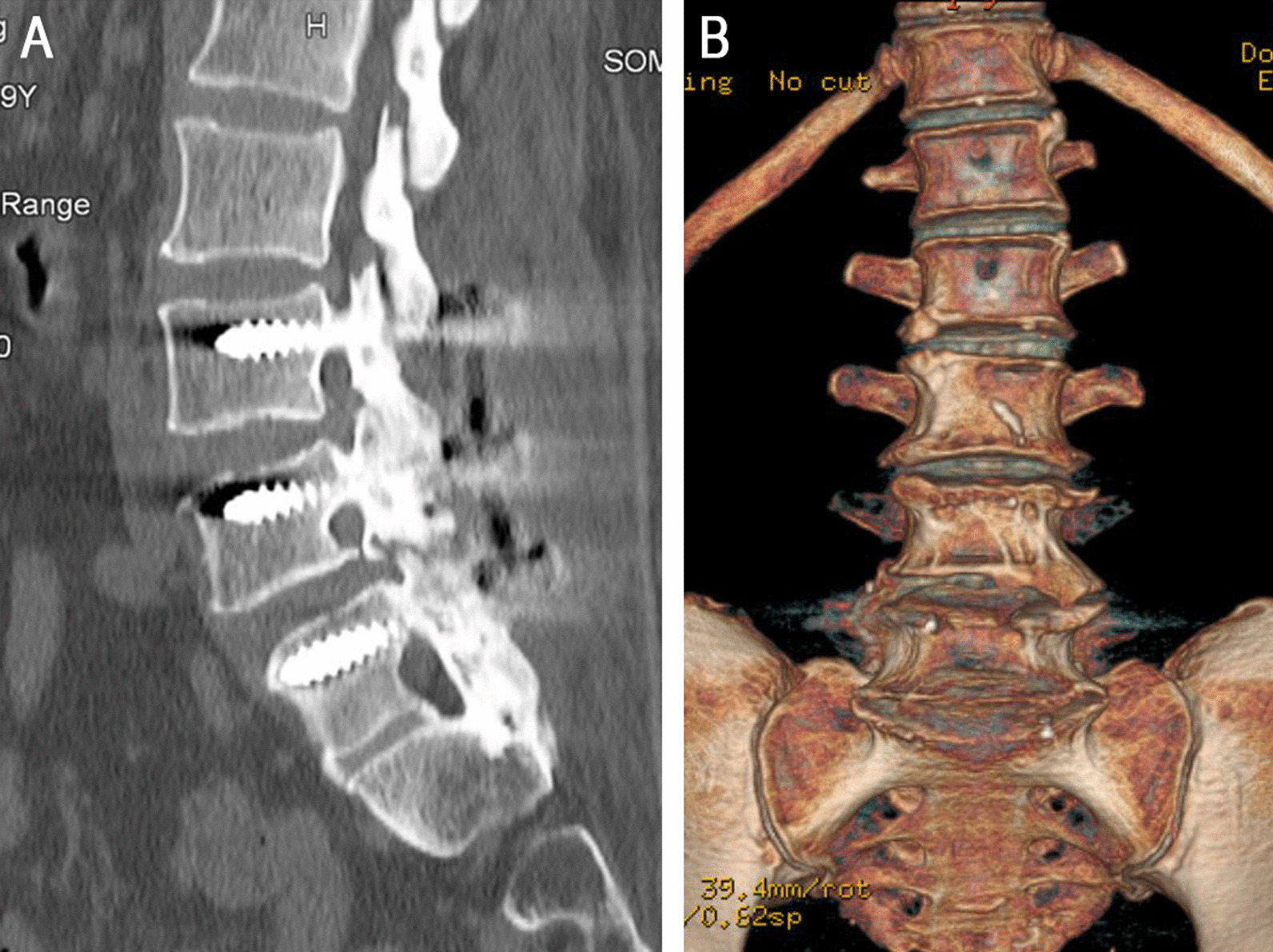
Sample views of lumbar computed tomography (CT) scan in the modified facet joint fusion (MFF) and posterolateral fusion (PLF) groups. **A** Lumbar CT scan with sagittal reconstruction views in the MFF group. **B** Lumbar CT scan with metal artifact reducing three-dimensional reconstruction views in the PLF group

The primary outcome was the fusion rate. Fusion status was evaluated by lumbar CT scan with sagittal reconstruction views in MFF group and with metal artifact reducing three-dimensional reconstruction views in PLF group 1 year after operation. The sample views was shown in Fig. 1. The fusion grade was classified according to the classification criteria showed in Table 2. Unilateral or bilateral grade I or II fusion was considered clinically fused, whereas grade III or IV fusion was considered unfused.

**Table 2:** Classification criteria for fusion assessment

Fusion Grade*	Radiographic criteria in MFF	Radiographic criteria in PLF
Grade I	Complete bony continuity covering a whole facet joint	Complete bony continuity in intertransverse area
Grade II	Partial bony continuity on a facet joint	Partial bony continuity in intertransverse area
Grade III	Bony continuity was not confirmed with certainty at any portion of a facet joint	Bony continuity was not confirmed with certainty in intertransverse area
Grade IV	Obvious nonunion of a facet joint	Obvious nonunion in intertransverse area

*Unilateral or bilateral grade I or II fusion was considered clinically fused, whereas grade III or IV fusion was considered unfused

Secondary outcomes were scores on the Oswestry Disability Index (ODI), the scores on the visual analog scale (VAS) for back pain and leg pain, the Japanese Orthopedic Association (JOA) score and the European Quality of Life–5 Dimensions (EQ-5D) score which were recorded preoperatively and 1 year postoperatively. The ODI score, which ranges from 0 to 100, with higher scores indicating more severe disability, is a standard index for measuring the degree of disability and estimating the quality of life in persons with lumbar degenerative disease. The VAS score, which range from 0 to 10, with higher scores indicating more severe pain. The JOA score, which ranges from 0 to 29, with lower scores indicating more severe disability. The EQ-5D score, which ranges from 0 to 1, with higher scores indicating better quality of life.

Moreover, information about the length of hospital stay, length of operation, intraoperative blood loss, cost of hospitalization, complications and reoperations was collected from the patients’ medical files.

### Statistical analysis

Statistical analysis was performed with SPSS software (SPSS version 24.0, SPSS Inc.). Statistical comparisons of the preoperative or postoperative ODI, VAS, JOA and EQ-5D scores between the two groups were performed using the nonparametric Wilcoxon-Mann–Whitney test. P < 0.05 was taken to indicate that the difference was statistically significant.

## Results

### Preoperative data

No significant differences were found in age, sex, or BMI score between the 2 groups (P > 0.05; Table 1). The preoperative ODI, VAS, JOA and EQ-5D scores from both groups are shown in Table 1; no significant differences were found between the 2 groups (P > 0.05). The detailed data for both groups are shown in Table 1.

### Evaluation results

In terms of fusion rates, according to the classification criteria, the fusion rate of MFF group was 94.3% (33/35), while the fusion rate of PLF group was 76.2% (32/42) 1 year after operation. The difference of the fusion rate was statistically significant (P < 0.05).

As measured by the ODI scores, the symptoms of the patients improved. The patients presented with functional limitations before surgical treatment, with ODI scores of 28.43 ± 13.82 in the PLF group and 24.00 ± 14.55 in the MFF group (P > 0.05), whereas 1 year after surgical treatment, the ODI score decreased to 8.93 ± 9.75 in the PLF group and 5.82 ± 5.29 in the MFF group (P > 0.05), which indicates improvements in the prior functional limitations and quality of life.

Analysis of the VAS scores for both lumbar and leg pain showed significant improvement during the postoperative period with respect to the preoperative period. There was no difference in the preoperative and postoperative VAS scores between the two groups (both P > 0.05).

The JOA score also indicated that the patients experienced a significant improvement in their symptoms. Before surgery, the JOA scores were 11.86 ± 3.74 in the PLF group and 11.91 ± 2.77 in the MFF group (P > 0.05), whereas 1 year after surgical treatment, the JOA scores were 13.57 ± 1.45 in the PLF group and 14.18 ± 2.27 in the MFF group (P > 0.05). The changes in the JOA scores also indicated significant improvement in the patients’ symptoms.

The quality of life, evaluated by the EQ-5D questionnaire, also showed a significant improvement 1 year after surgery. The evaluation results described above for both groups are shown in Table 3.

**Table 3:** Results of the evaluate questionnaires

Variable	PLF group (N = 42)	MFF group (N = 35)	P value	Statistical power
ODI score	8.93 ± 9.75	5.82 ± 5.29	0.572	0.513
VAS score for lumbar pain	2.07 ± 2.79	3.18 ± 3.25	0.244	0.462
VAS score for leg pain	1.86 ± 2.57	2.45 ± 3.17	0.687	0.218
JOA score	13.57 ± 1.45	14.18 ± 2.27	0.647	0.386
EQ-5D score	0.88 ± 0.17	0.93 ± 0.79	0.809	0.101

### Perioperative data

During the perioperative period, we also analyzed the length of hospital stay, length of operation, intraoperative blood loss, and cost of hospitalization. Regarding hospital stay, the average duration was 12.5 ± 2.3 days in the PLF group and 11.5 ± 4.4 days in the MFF group (P > 0.05). In terms of the length of operation, the average was 168.5 ± 52.4 min in the PLF group and 131.7 ± 29.6 min in the MFF group (P > 0.05). Regarding intraoperative blood loss, the amounts were 335.0 ± 205.5 ml in the PLF group and 156.7 ± 75.3 ml in the MFF group; the difference was statistically significant (P < 0.05). Regarding the cost of hospitalization, the average was 81,361.4 ± 16,768.6 yuan in the PLF group and 78,492.0 ± 10,615.9 yuan in the MFF group (P > 0.05). The detailed results for both groups are shown in Table 4.

**Table 4:** Perioperative information

Variable	PLF group (N = 42)	MFF group (N = 35)	P value	Statistical power
Length of hospital stay (d)	12.5 ± 2.3	11.5 ± 4.4	0.428	0.330
Length of operation (min)	168.5 ± 52.4	131.7 ± 29.6	0.062	0.978
Intraoperative blood loss (ml)	335.0 ± 205.5	156.7 ± 75.3	0.003	0.998
Cost of hospitalization (¥)	81,361.4 ± 16,768.6	78,492.0 ± 10,615.9	0.643	0.218

### Complications

Complications during the perioperative period and follow-up period were as follows. In the PLF group, there was 1 patient with poor wound healing, who healed well after debridement. Three patients with intermuscular vein thrombosis recovered well after standard anticoagulant therapy. Two patients with urinary tract infection recovered well after antibiotic treatment. In the MFF group, there were 2 patients with intermuscular vein thrombosis, who recovered well after anticoagulant treatment. The urinary tract infections of 2 patients recovered well after antibiotic treatment. No iatrogenic nerve injury, vascular injury, epidural hematoma, intravertebral infection, or internal fixation-related complications occurred in either group. None of the patients required reoperation.

## Discussion

Patients with lumbar degenerative disease typically present with low back pain and leg pain, which occur especially when they are walking. This degenerative condition severely restricts function, walking ability, and quality of life [16, 17]. These lumbar symptoms have become the most common indication for spinal surgery, and studies have shown that surgical treatment in selected patients is more successful than conservative alternatives [18–20].

In this study, we described a modified facet joint fusion technique for the treatment of lumbar degenerative disease. In this technique, the scope of lamina excision and the method for fabricating the bone graft bed is very important. Traditional facet joint fusion uses a bone chisel to shape the joint space into a “V” shape. This bone graft bed has a small area, and the quality of the resulting graft cannot be guaranteed. In our technique, a high-speed grinding drill with a diameter of approximately 3 mm is used to remove the cartilage and bone cortex in the joint space, thus creating a “U”-shaped graft bed, which not only ensures the quality of the graft but also greatly increases the area of the bone graft bed. At the same time, it avoids the defect of creating joint process fracture to a large extent, which could be easily created by the traditional fusion technique. Different bone graft materials, such as autogenous cancellous bone and allogeneic cancellous bone granules, could be implanted into the bone graft bed. In this study, the VAS scores for lumbar pain and leg pain, the ODI, and the JOA and EQ-5D scores were significantly improved after surgery, and there was no significant difference in the treatment effect between the PLF and MFF techniques, and no complications related to MFF occurred. Therefore, we believe that MFF is a safe and convenient fusion technique, with a therapeutic effect no worse than that of PLF.

On surgical indications, MFF and PLF are feasible for patients with lumbar disc herniation and lumbar spinal stenosis combined with symptoms, such as lumbar and leg pain, paresthesia, and muscle loss caused by lumbar degenerative diseases. MFF can be considered for patients without lumbar spondylolisthesis above grade I, spondylolysis, scoliosis, and lumbar fracture.

This study selected all patients who met the inclusion and exclusion criteria during the specific period to minimize selection bias. Excluding patients with long-segment lumbar surgery and more severe lumbar degenerative disease also causes selection bias. It is good practice to obtain results from the minimally adjusted available model.

In 1999, Park et al. [21] described facet joint fusion techniques and reported a fusion rate of 93.8% in patients with degenerative lumbar spondylolisthesis (n = 32) followed up for more than 1 year. However, in their research, the bone graft material was autogenous iliac cancellous bone. Although autologous bone is the most ideal bone graft material, the procedure used to acquire it may lead to pain, bleeding, infection and other complications [22]. In our technique, autologous cancellous bone was extracted from the intraoperative spinous process and lamina, supplemented by allogeneic cancellous bone particles, which not only avoids the above complications but also achieves the same therapeutic effect. However, it has not been widely used in clinical practice.

In 2015, Miyashita et al. [11] studied facet joint fusion in patients with degenerative lumbar scoliosis (n = 88) and achieved a fusion rate of 88%. The production criterion for the bone graft bed was a length or depth of at least 1 cm, which we thought was not very accurate. Because most of the facet joints in patients with lumbar degenerative disease show obvious proliferative sclerosis, the quality of the bone graft bed cannot be guaranteed simply by making the bone graft bed according to a standard of length or depth. In our research, we used a high-speed grinding drill to remove the cartilage and cortical bone in the facet joint space until the cancellous bone surface was exposed to ensure the quality of the bone graft bed.

In previous studies, the fusion rate of PLF ranges from 72.3 to 90% [1, 23–25]. In our study, the fusion rate was 76.2%, which was similar with the result in the other studies. PLF is a quite widely used surgical method to treat lumbar degenerative disease. However, because of the need to expose paraspinal muscles, patients are more likely to experience postoperative low back pain compared with the other surgical methods [11]. In our study, the fusion rate of MFF was significantly higher than PLF and the treatment effect was similar between the two groups. Therefore, for short-segment lumbar degenerative disease, MFF should be a better choice compared with PLF.

Because the gap between the facet joints is narrow and the bone graft is firmly in contact with the surrounding bone, theoretically, the fusion rate is higher. In the future, MFF can be used in patients with the aforementioned surgical indications and has broad clinical applications. If it can be widely performed in clinical practice, it will reduce patients' surrounding tissue injury and improve the fusion rate. As for mechanical strength, solid fusion can effectively reduce the stress of the internal fixation system. Thus, it reduces the incidence of internal fixation fatigue fractures, prevents mechanical failure, and ensures long-term postoperative effects. There is still a lack of relevant studies on whether there is a difference in spinal stability after MFF and PLF. Presently, no complications, such as internal fixation fracture, were observed in the one-year follow-up. However, systematic biomechanical studies and long-term clinical efficacy and complications still need further study.

In our study, there was no significant difference between the MFF group and the PLF group in terms of both the ODI and the VAS, JOA, or EQ-5D scores and in the length of hospital stay, length of operation, intraoperative blood loss, cost of hospitalization, and perioperative complications, indicating that the treatment effect for lumbar degenerative diseases in the MFF group was no worse than that in the PLF group. In addition, the intraoperative blood loss of the MFF group was significantly lower than that of the PLF group, which may be related to the fewer injuries to the paravertebral muscle, the smaller wound surface and the relatively shorter operation time in the MFF group than in the PLF group. If the modified facet joint fusion technique can be widely used in lumbar surgery, the intraoperative blood loss of patients will be effectively reduced. According to our study, the MFF technique can be used for lumbar fusion in patients with degenerative lumbar diseases undergoing short-segment surgery without severe lumbar spondylolisthesis, scoliosis, spondylolysis, and lumbar fracture.

Compared with the currently widely used intervertebral fusion, the advantage of modified facet joint fusion is that the latter technique is simple and does not need to be performed on the anterior column or middle column, so it can effectively avoid complications associated with intervertebral fusion [6, 26]. In this study, no complications related to the MFF technique occurred. In addition, the modified facet joint fusion technique does not require special equipment, such as cages, so the cost to the patient should be reduced. However, a detailed comparison between modified facet joint fusion and intervertebral fusion requires further study and analysis.

This study has the following limitations. First, we only evaluated the fusion rate at 1 year postoperatively and did not assess changes of fusion rates dynamically. Second, the 1 year for the postoperative follow-up is relatively short, so it is necessary to further extend the follow-up duration to evaluate the long-term effectiveness of the treatments. Third, we only selected patients with 1–2 levels of lumbar degenerative disease for evaluation. In further study, patients with multiple levels of lumbar degenerative disease can be included.

## Conclusion

In terms of different evaluation indexes, there was no significant difference in therapeutic effect between the two groups. The effect of the modified facet joint fusion technique in the treatment of degenerative lumbar diseases was no worse than that of posterolateral fusion. However, the fusion rate of MFF is significantly higher than PLF. At the same time, MFF results in lighter surgical trauma and less intraoperative blood loss than PLF. Therefore, the MFF technique, with simple technical requirements, acceptable safety and relatively little blood loss, is worthy of promotion in clinical practice.

## Data Availability

The datasets used and/or analysed during the current study available from the corresponding author on reasonable request.
